# Local, national, and regional viral haemorrhagic fever pandemic potential in Africa: a multistage analysis

**DOI:** 10.1016/S0140-6736(17)32092-5

**Published:** 2017-12-16

**Authors:** David M Pigott, Aniruddha Deshpande, Ian Letourneau, Chloe Morozoff, Robert C Reiner, Moritz U G Kraemer, Shannon E Brent, Isaac I Bogoch, Kamran Khan, Molly H Biehl, Roy Burstein, Lucas Earl, Nancy Fullman, Jane P Messina, Adrian Q N Mylne, Catherine L Moyes, Freya M Shearer, Samir Bhatt, Oliver J Brady, Peter W Gething, Daniel J Weiss, Andrew J Tatem, Luke Caley, Tom De Groeve, Luca Vernaccini, Nick Golding, Peter Horby, Jens H Kuhn, Sandra J Laney, Edmond Ng, Peter Piot, Osman Sankoh, Christopher J L Murray, Simon I Hay

**Affiliations:** a Institute for Health Metrics and Evaluation, University of Washington, Seattle, WA, USA; b Department of Zoology, University of Oxford, Oxford, UK; c School of Geography and the Environment, University of Oxford, Oxford, UK; d School of Interdisciplinary Area Studies, University of Oxford, Oxford, UK; e Big Data Institute, Li Ka Shing Centre for Health Information and Discovery, University of Oxford, Oxford, UK; f Centre for Tropical Medicine and Global Health, Nuffield Department of Medicine, University of Oxford, Oxford, UK; g Harvard Medical School, Harvard University, Boston, MA, USA; h Boston Children's Hospital, Boston, MA, USA; i Li Ka Shing Knowledge Institute, St Michael's Hospital, Toronto, ON, Canada; j Divisions of General Internal Medicine and Infectious Diseases, Toronto General Hospital, Toronto, ON, Canada; k Department of Medicine, University of Toronto, Toronto, ON, Canada; l Warwick Medical School, University of Warwick, Coventry, UK; m Department of Infectious Disease Epidemiology, Imperial College London, London, UK; n Department of Infectious Disease Epidemiology, London School of Hygiene & Tropical Medicine, London, UK; o Director's Office, London School of Hygiene & Tropical Medicine, London, UK; p WorldPop, Department of Geography and Environment, University of Southampton, Southampton, UK; q Flowminder Foundation, Stockholm Sweden; r The Start Network, London, UK; s European Commission, Joint Research Centre, Ispra, Italy; t Quantitative and Applied Ecology Group, School of BioSciences, University of Melbourne, Parkville, VIC, Australia; u Integrated Research Facility at Fort Detrick, Division of Clinical Research, National Institute of Allergy and Infectious Diseases, National Institutes of Health, Frederick, MD, USA; v SJL Global Consulting, Seattle, WA, USA; w INDEPTH Network, Accra, Ghana

## Abstract

**Background:**

Predicting when and where pathogens will emerge is difficult, yet, as shown by the recent Ebola and Zika epidemics, effective and timely responses are key. It is therefore crucial to transition from reactive to proactive responses for these pathogens. To better identify priorities for outbreak mitigation and prevention, we developed a cohesive framework combining disparate methods and data sources, and assessed subnational pandemic potential for four viral haemorrhagic fevers in Africa, Crimean–Congo haemorrhagic fever, Ebola virus disease, Lassa fever, and Marburg virus disease.

**Methods:**

In this multistage analysis, we quantified three stages underlying the potential of widespread viral haemorrhagic fever epidemics. Environmental suitability maps were used to define stage 1, index-case potential, which assesses populations at risk of infection due to spillover from zoonotic hosts or vectors, identifying where index cases could present. Stage 2, outbreak potential, iterates upon an existing framework, the Index for Risk Management, to measure potential for secondary spread in people within specific communities. For stage 3, epidemic potential, we combined local and international scale connectivity assessments with stage 2 to evaluate possible spread of local outbreaks nationally, regionally, and internationally.

**Findings:**

We found epidemic potential to vary within Africa, with regions where viral haemorrhagic fever outbreaks have previously occurred (eg, western Africa) and areas currently considered non-endemic (eg, Cameroon and Ethiopia) both ranking highly. Tracking transitions between stages showed how an index case can escalate into a widespread epidemic in the absence of intervention (eg, Nigeria and Guinea). Our analysis showed Chad, Somalia, and South Sudan to be highly susceptible to any outbreak at subnational levels.

**Interpretation:**

Our analysis provides a unified assessment of potential epidemic trajectories, with the aim of allowing national and international agencies to pre-emptively evaluate needs and target resources. Within each country, our framework identifies at-risk subnational locations in which to improve surveillance, diagnostic capabilities, and health systems in parallel with the design of policies for optimal responses at each stage. In conjunction with pandemic preparedness activities, assessments such as ours can identify regions where needs and provisions do not align, and thus should be targeted for future strengthening and support.

**Funding:**

Paul G Allen Family Foundation, Bill & Melinda Gates Foundation, Wellcome Trust, UK Department for International Development.

## Introduction

The Ebola virus disease outbreak, which centred in Guinea, Liberia, and Sierra Leone, was unprecedented both in terms of mortality and morbidity, as well as the extent to which the disease spread locally and internationally.^1^ The unanticipated cases of Ebola virus disease in regions previously considered non-endemic, coupled with inadequate infrastructure and susceptible, yet highly mobile populations, might have contributed to the outbreak infecting over 60 times more individuals than any previous Ebola virus disease outbreak.^2^ As pathogens continue to emerge and spread into populations at-risk, a move from purely responsive activities to also include proactive management of emerging infectious diseases is urgently needed.^3^, ^4^ The current paradigm of responding to these threats as and when they arise is expensive and unsustainable.^5^ Initiatives such as the Coalition for Epidemic Preparedness Innovations and the US Global Health Security Agenda, with its renewed focus on achieving the International Health Regulations, have reinforced a need for a proactive approach to emerging infectious diseases.^6^ Consequently, there is great interest in the development of tools to help pre-empt such outbreaks and inform broad-scale health-system strengthening with respect to emerging infectious diseases,^7^, ^8^ and particularly, to establish the prioritisation of limited resources^9^ and the optimal deployment of surveillance, preventive measures, and treatments.

**Research in context**

**Evidence before this study**

We searched PubMed with search terms “viral haemorrhagic fever preparedness”, “viral haemorrhagic fever risk assessment”, “pandemic risk assessment”, and “pandemic preparedness”, for articles published between Jan 1, 1990, and July 1, 2016, with supplemental searches in Google Scholar. Articles assessing subnational variation in pandemic risk or evaluating this risk across broad geographic scales were included. A variety of analytical approaches have been developed to assess different aspects of outbreak risk, including environmental correlates to define regions of possible pathogen emergence, models of population connectivity to determine potential spread, and analyses of drivers of incidence and prevalence at local and national levels. Most of these assessments are retrospective, or delayed due to time lags in data availability and hence may have limited utility for improving epidemic preparedness. Pre-emptive assessments of epidemics, leveraging common features from such work are less common. Hotspots of disease emergence have been identified and indices developed to gauge national-level susceptibility to infectious pathogens, representing important first steps in assessing risk. To date, however, no analyses have quantified how these vulnerabilities might change during different stages of an outbreak, nor have they consistently evaluated such vulnerabilities for the spatial granularity at which outbreaks occur and are addressed.

**Added value of this study**

This study builds upon previous preparedness concepts to develop a subnational evaluation of epidemic potential across Africa. For the first time, within a single pandemic potential framework, our analyses show how different stages of an outbreak can be quantified and evaluated before the next outbreak. We use a new combination of methods to provide a complete picture of potential outbreak progression, from initial spillover resulting in an index case to broader regional and international spread; thus allowing countries to directly focus on places where disease emergence and subsequent transmission could have the greatest impact on human health.

**Implications of all the available evidence**

Our results allow both governmental and non-governmental organisations to better understand epidemic potential, and offer pre-emptive planning to address vulnerabilities before the next outbreak of viral haemorrhagic fever. Using these results in tandem with existing in-country evaluations, such as the Joint External Evaluations, allows stakeholders to establish whether outbreak preparedness activities, broader governmental initiatives, or pathogen-specific control measures, are being targeted appropriately to places with future outbreak potential. Regions where there is a mismatch between pandemic potential and existing preparedness activities should therefore be considered as priorities in future preparedness planning. By moving preparedness discussions from national to subnational assessments, outbreak response protocols can better reflect the demands that epidemics present to local health systems.

Several studies have identified pathogens with pandemic potential and key drivers of their distribution and emergence,^10^, ^11^ as well as defined vulnerable countries.^12^ Further, various methods and technologies can be used once cases are reported, aiding the response, tracking, and predicting progression from an index case to widespread epidemic.^13^, ^14^, ^15^, ^16^ However, few systems are in place to synthesise these disparate analyses in a consolidated framework that outlines this potential progression,^17^, ^18^ and no work to date, to our knowledge, accounts for subnational variations that more closely reflect the geographic level at which outbreaks occur.

Viral haemorrhagic fevers, such as Crimean–Congo haemorrhagic fever, Ebola virus disease, Lassa fever, and Marburg virus disease, are present in Africa and have the potential for secondary human-to-human transmission after zoonotic spillover into human populations, with initial clinical presentation similar to several other pathogens. Subsequently, they pose a risk to populations in both endemic and non-endemic locations, particularly where rapid diagnostic capacity is low.^19^

**Figure 1:**
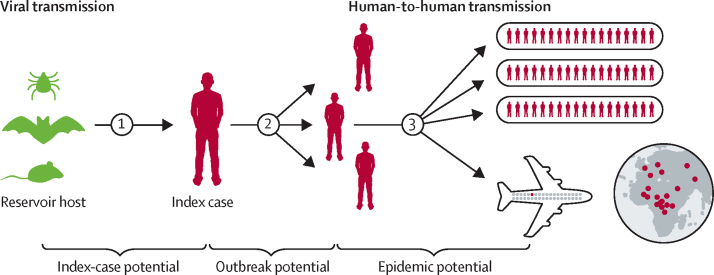
Conceptual progression of a viral haemorrhagic fever from animal reservoir to global pandemic Keys stages in the progression to a potential widespread epidemic are summarised. Stage 1, index-case potential, refers to spillover viral transmission from animal reservoir to index cases. Stage 2, outbreak potential, represents an index case infecting individuals within the local community or in a care-giving setting quantified via a composite indicator assessing outbreak receptivity. Stage 3, epidemic potential, reﬂects the widespread transmission of the virus both at regional and international scales.

To assess the ability of these pathogens to cause a widespread epidemic, we developed a framework that focuses on key transition points in a potential outbreak. By addressing different stages of an epidemic, we aim to provide actionable information on where to focus existing countermeasures. Additionally, by using a subnational unit of reference, we aim to provide information at a scale comparable to the localised nature of outbreaks, allowing for identification of communities at greatest risk. Drawing from multiple data sources and methods, this study outlines a three-stage framework (figure 1): stage 1, index-case potential, describes the transition (spillover) from zoonotic reservoirs or vectors into human populations, resulting in an index case (ie, the first case in any potential epidemic); stage 2, outbreak potential, characterises the subsequent secondary spread of the pathogen in people, typically localised where care is given, whether at home or in the health-care system and nearby settlements; stage 3, epidemic potential, describes the processes by which local outbreaks can subsequently cause infections elsewhere nationally, regionally, and internationally.

## Methods

### Overview

In this multistage analysis, we characterised three stages of a potential viral haemorrhagic fever outbreak with a variety of methods and datasets (table). We used environmental suitability maps to define at-risk populations for stage 1. Stage 2 is an adaptation of an existing risk framework, the Index for Risk Management (INFORM),^44^, ^45^ and uses a composite indicator to assess variation in outbreak receptivity (ie, locations where continued transmission in people is more likely to occur) combined with stage 1 estimates to identify regions with the highest potential for localised outbreaks. For stage 3, we combine connectivity assessments, both at the local and international scale, with stage 2 to establish regions with the highest potential for widespread epidemics.

**Table:** Input datasets used in the pandemic potential framework

		**Resolution**	**Data source**
**Stage 1 index case potential**			
Crimean–Congo haemorrhagic fever: environmental suitability		5 × 5 km	Messina et al^20^
Crimean–Congo haemorrhagic fever: occurrence records		Geopositioned records	Messina et al^21^
Ebola virus disease: environmental suitability		5 × 5 km	Pigott et al^22^
Ebola virus disease: occurrence records		Geopositioned records	Mylne et al^23^
Lassa fever: environmental suitability and occurrence records		5 × 5 km	Mylne et al^24^
Marburg virus disease: environmental suitability and occurrence records		5 × 5 km	Pigott et al^25^
Population		5 × 5 km	WorldPop^26^
**Stage 2 outbreak potential**			
Governance			
	Government effectiveness	National	World Bank^27^
	Corruption perception index*	National	Transparency International^28^
	Donor aid	National	IHME^29^
	Conflict	Subnational	ACLED^30^
Communications			
	Educational attainment	National	IHME^31^
	Internet	National	World Bank^32^
	Cellular phone subscriptions	National	World Bank^33^
	Electricity	National	World Bank^34^
Isolation			
	Proportion rural	5 × 5 km	GRUMP^35^
	Travel time to nearest major settlement	5 × 5 km	Nelson^36^
Infrastructure			
	Access to improved water	National and subnational	GAHI^37^ and IHME^38^
	Access to improved sanitation	National and subnational	GAHI^37^ and IHME^38^
Health care			
	DPT3 coverage	National	WHO^39^
	Lower respiratory infections*	National	IHME^40^, ^41^
	Diarrhoeal disease*	National	IHME^40^, ^41^
	Health-care expenditure as a percentage of GDP	National	WHO^42^
	Under-5 mortality	5 × 5 km	Golding et al^43^
**Stage 3 epidemic potential**			
Travel time to nearest major settlement		5 × 5 km	Nelson^36^
Outbound passenger volume (flights)		National	IATA†

The table outlines covariates included for each stage, and their provenance. In stage 2, each component is broken down into its constituent factors: governance, communications, isolation, infrastructure, and health care. IHME=Institute for Health Metrics and Evaluation. ACLED=Armed Conflict Location & Event Data Project. GAHI=Global Alliance for Humanitarian Innovation. GRUMP=Global Rural-Urban Mapping Project. DPT3=diphtheria-tetanus-pertussis. GDP=gross domestic product. IATA=International Air Transport Association.

* Excluded after redundancy analysis.

† Data not publicly available.

We assessed information at the second national administrative division (admin 2) as defined by the UN Food and Agriculture Organization.^46^ Following INFORM protocols, we normalised covariate factors and standardised to a 0–10 output, with 10 representing the worst outcome. Contingent on the potential for compensatory effects between factors, we used either geometric or arithmetic means when aggregating data, with each stage building on the previous stage. We established uncertainty by calculating 1000 draws, each using independent random draws for covariates with associated variance. We assessed uncertainty at each of the three stages. Aggregation of the results for all stages occurs after this process, ensuring a full record of the uncertainty by stage. Importantly, the output measures of median ranks allow for comparison, but are not direct representations of quantitative differences in potential case numbers and deaths.

This study follows the Guidelines for Accurate and Transparent Health Estimates Reporting (GATHER).^47^ Further details on the estimation and data sources used in this analysis are included in the supplementary materials, and all code used for these analyses are available on request from the corresponding author.

### Stage 1: assessing index-case potential based on environmental suitability

We used existing models of environmental suitability for the transmission of a virus from environmental sources into human populations to establish regions at risk of spillover infections.^20^, ^22^, ^24^, ^25^, ^48^ These maps use reported geographic information on index cases of outbreaks and viral detection in animals,^21^, ^23^, ^24^, ^25^ which are related to environmental drivers using species distribution models.^49^, ^50^ We compiled this information to build an environmental profile that best characterises possible pathogen presence. Subsequently, areas of unknown disease status can be evaluated based on their environmental profile similarities. We calculated a data-driven threshold value defining at-risk areas by assessing different groupings of reported disease occurrences and background records, and the ability of each threshold to accurately classify them.^51^, ^52^ This process was repeated 1000 times. Total population count evaluated at a 5 × 5 km resolution,^53^ and the proportion of the total administrative unit population living within these locations, were aggregated to the admin 2 level^46^ and standardised on a scale of 0–10.^44^ We evaluated a final stage 1 value by calculating an inverted geometric mean of these two population measures.^44^ More detail on the conversion of these niche maps and quantification of populations at risk is provided in the supplementary materials.

### Stage 2: quantifying outbreak potential

We paired stage 1 estimates of index-case potential with a composite indicator of measures termed outbreak receptivity (table) to produce admin 2 level outbreak potential assessments, identifying locations more likely to experience secondary human-to-human viral transmission. We established an initial set of measures, referred to here as factors, through expert consultation based on the INFORM study,^44^ and revised for relevance to infectious diseases through a meeting at UK Department for International Development. We identified factors and grouped them into five key components: governance,^27^, ^28^, ^29^, ^54^ communications,^31^, ^32^, ^33^, ^34^ isolation,^35^, ^36^ infrastructure,^37^, ^38^ and health care,^39^, ^40^, ^41^, ^42^, ^43^ based on hypothesised similar effects on outbreaks. This outbreak receptivity indicator and its components are intended to reflect the susceptibility of a given location to continued transmission based on the resident population and existing response infrastructure. Thus, each included factor is a hypothesised correlate of continued secondary transmission.

Other factors such as access to personal protective equipment or number of isolation wards were considered, but were ultimately excluded because data at a continental scale were not available. We collected information on each factor at the highest possible spatial resolution and summarised data at the admin 2 level. We standardised inputs to a 0–10 scale, including normalisation where appropriate, using optimised Box-Cox transformations,^55^ and input variation simulated by drawing values from uncertainty estimates or data time series (supplementary materials). We took 1000 draws for each admin 2 per factor and aggregated into respective components, and then the composite indicator (table). Initial aggregation weighed components equally. In testing this composite indicator, we identified uniqueness among factors via principal components analysis, and removed redundant factors (ie, those with similar scores across the first two principal components).^44^ We calculated variance-based importance metrics for sensitivity to analyse unintentional dominance of any one component.^56^, ^57^ Given that a subset of all possible outbreak drivers are used with no a priori rationale for one component being dominant, we enforced equivalence by reweighting the arithmetic mean so that the differences in variance explained of the final composite indicator by each component were reduced. We coupled the resultant outbreak receptivity values with stage 1 outputs for each pathogen by taking their geometric mean, to produce the final stage 2 evaluation.

### Stage 3: estimating epidemic potential based on local and international connectivity

We evaluated two dimensions of epidemic potential for stage 3. First, we assessed the source capacity of an admin 2 unit (ie, the potential for an outbreak to seed infections in other locations) (stage 3a) by measuring the average travel time from a given 5 × 5 km unit in each admin 2 to the nearest city (defined as over 50 000 inhabitants).^36^ With this calculation, we identified the at-risk locations most likely to be exporters of infected individuals, in the absence of barriers to movement such as border restrictions. The travel time covariate uses information on land cover and existing infrastructure to estimate the shortest travel time from any given point to nearby settlements. We normalised this covariate and standardised to a 0–10 scale, and the geometric mean of stage 2 outputs and this covariate were taken to produce the measure for stage 3a. Second, we assessed international source capacity (stage 3b) with anonymised passenger-level flight itineraries from the International Air Transport Association for 2015, which included details on passengers' initial airport of embarkation and final destination.^14^, ^58^ We produced the estimate for epidemic potential by pairing national patterns of outbound passenger volume to any other global destination, with stage 2 outputs using geometric means.

### Role of the funding source

The funders of the study had no role in study design, data collection, data analysis, data interpretation, or writing of the report. The corresponding author had full access to all the data in the study and had final responsibility for the decision to submit for publication.

## Results

**Figure 2:**
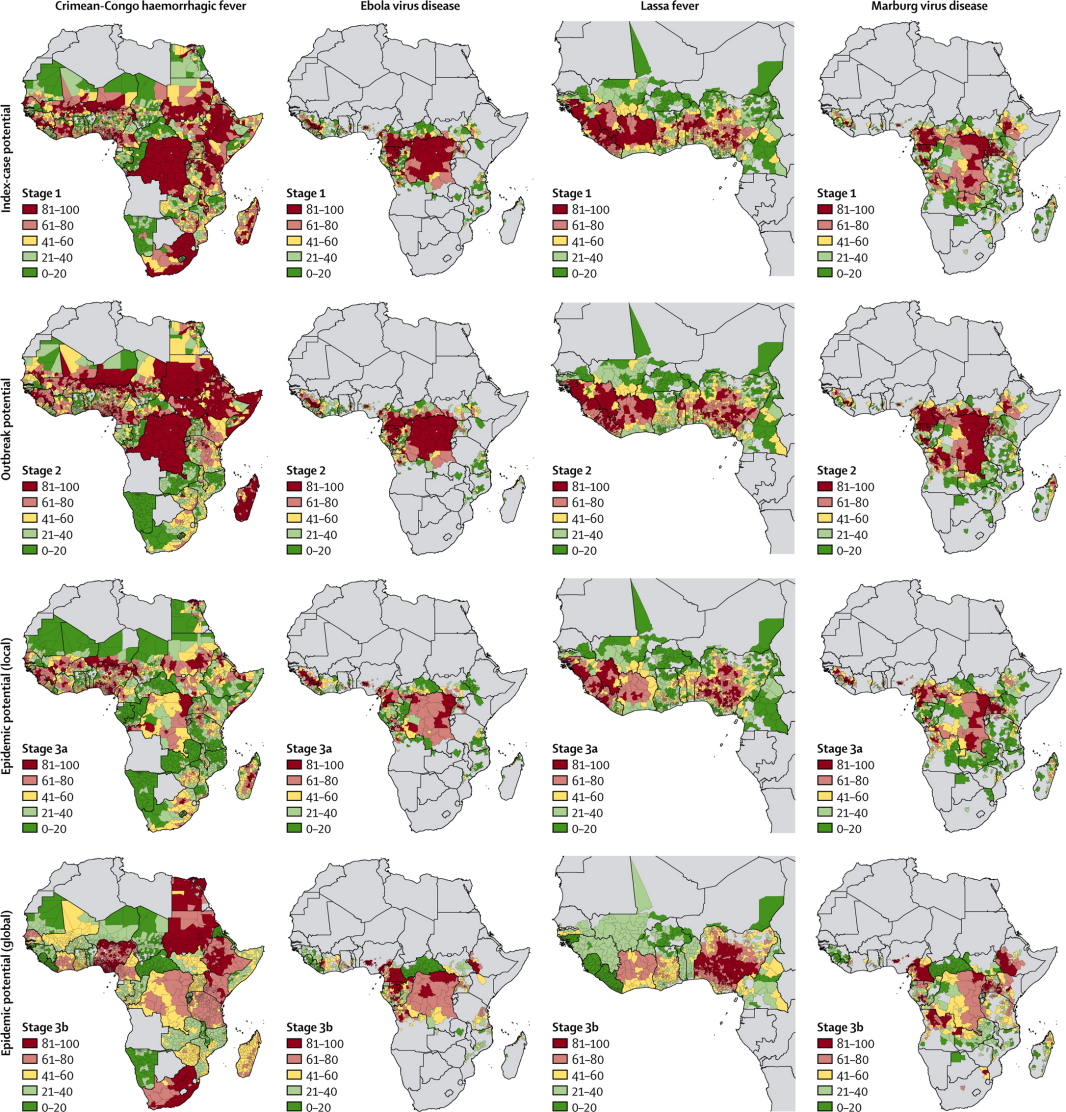
Pandemic potential of four African viral haemorrhagic fevers Each column represents the various stages of a potential pandemic, from initial index-case potential (first row) and outbreak potential (second row) to local epidemic potential (third row) and global epidemic potential (fourth row). Columns, moving from left to right, show this progression for Crimean–Congo haemorrhagic fever, Ebola virus disease, Lassa fever, and Marburg virus disease. For each figure, administrative units coloured in red are those with median values (based on 1000 draws) that rank in the top quintile of ranked units; units in dark green have median values that rank in the lowest quintile. Interactive maps are available via the online visualisation tools.

Here, we provide estimates for each at-risk subnational admin 2 in Africa across the three stages of a potential outbreak (figure 2). Interactive maps are made available via online visualisation tools.

**Figure 3:**
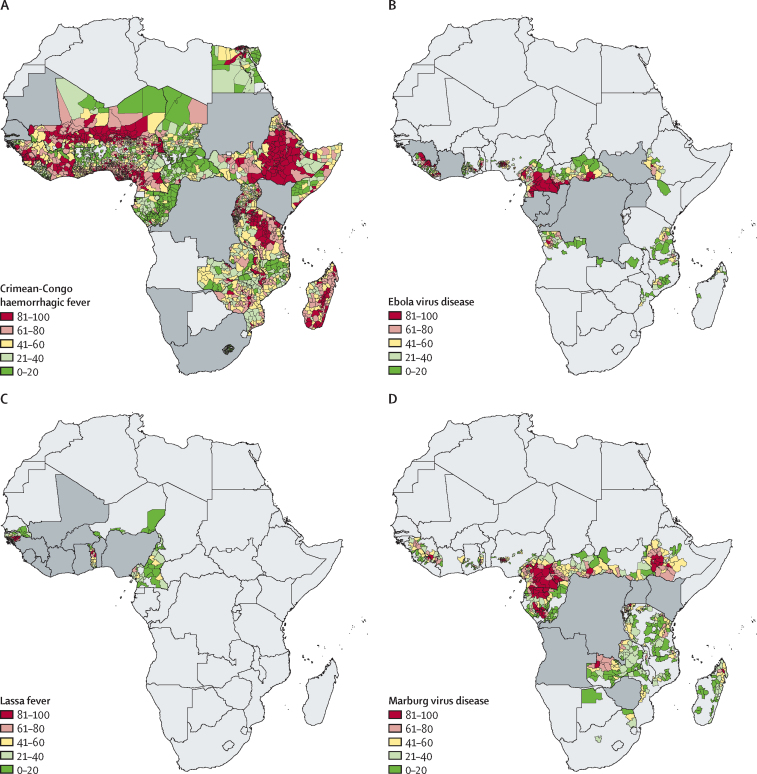
Index-case potential across countries that did not previously report spillover events Stage 1 index-case potential masked by previous reporting of index cases for Crimean–Congo haemorrhagic fever (A), Ebola virus disease (B), Lassa fever (C), and Marburg virus disease (D). Countries in dark grey are those that have previously seen spillover index cases reported. The remaining at-risk administrative units coloured in red are those with median values (based on 1000 draws) that rank in the top quintile of ranked remaining units; those coloured in green have median values that rank in the lowest quintile.

Across Africa, the ranking of subnational regions in stage 1, identification of locations with the greatest potential for index cases, reflected key trends observed in the original zoonotic niche maps (figure 2, first row). All admin 2 areas are ordered so that regions with the highest index-case potential are highest ranked. Areas susceptible to spillover of Crimean–Congo haemorrhagic fever were widespread, with the highest ranked admin 2 units found in the Sahel (eg, Kollo, Niger), the Horn of Africa (eg, Sennar, Sudan), and southern Africa (eg, Johannesburg, South Africa). By contrast, Lassa fever was restricted to areas in western Africa such as in Guinea (eg, Guéckédou) and Nigeria (eg, Ife North), whereas Ebola virus disease had highest ranked admin 2 units, and thus those with the highest potential for index cases, found in both western Africa (eg, Macenta, Guinea and Foya, Liberia) and middle Africa (eg, Woleu, Gabon and Haut-Uele, the Democratic Republic of the Congo). Highest ranked locations for Marburg virus disease were present across the continent (eg, Mwenge, Uganda; Voinjama, Liberia; and Beni, the Democratic Republic of the Congo). Although many regions in the highest quintile were located in countries with previous outbreaks, several locations with no previous outbreaks had high index-case potential, such as Boumba-et-Ngoko in Cameroon for Ebola virus disease (figure 3).

**Figure 4:**
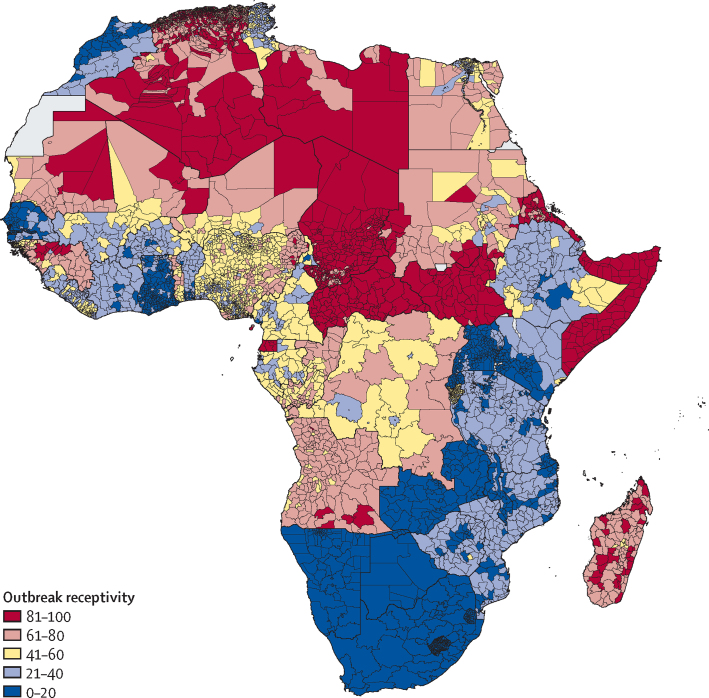
Outbreak receptivity The map displays the final outbreak receptivity indicator, a component in the stage 2 evaluation. Administrative units coloured in red are those with median values (based on 1000 draws) that rank in the top quintile of ranked units; those in dark blue have median values that rank in the lowest quintile. Interactive maps are available via the online visualisation tools.

Outbreak receptivity, the composite indicator used in stage 2 to identify regions susceptible to ongoing secondary transmission, revealed substantial variation in capabilities to effectively respond to index cases (figure 4). At least 90% of the districts found in the Central African Republic, Chad, Somalia, and South Sudan ranked in the top 90th percentile of African locations, or in other words, those administrative units with the most susceptible populations and poorest response capacity. More than 80% of admin 2 units in Guinea, Madagascar, and Sudan were in the top 70th percentile or higher for outbreak receptivity.

Pairing outbreak receptivity (stage 2) with index-case potential (stage 1) highlights key differences in pathogen-specific vulnerabilities between locations (figure 2, second row) which shows that some regions are more susceptible to continued human-to-human transmission. Locations rising up the rankings between stages indicate that, in the event of an index case, these would be key targets for rapid and effective intervention, to avert local outbreaks. For Crimean–Congo haemorrhagic fever, many at-risk districts increased in rankings, particularly in Somalia (54% increase in districts in top quintile admin 2) and across the Sahel, such as within Sudan (+25%) and Niger (+14%). Conversely, rankings decreased between stages in all districts in South Africa (85% reduction in top quintile admin 2) as well as parts of Kenya (−51%) and Tanzania (−29%). Similar patterns were observed with Ebola virus disease, with locations in Angola (+14%), the Central African Republic (+7%), and South Sudan (+7%) ranking higher, whereas districts in Côte d'Ivoire (−5%) and Uganda (−19%) decreased between stages. By contrast with Ebola virus disease, Lassa fever showed variable changes in ranking across western Africa; Guinean districts rose in ranking (+15%), Ivorian districts decreased (−28%), and Nigerian districts reflected the heterogeneities in receptivity found across the country. For Marburg virus disease, rankings rose most in at-risk districts in Angola (+13%) and the Democratic Republic of the Congo (+21%), whereas similar national-level trends occurred in line with changes seen in Ebola virus disease rankings (eg, 35% reduction in top quintile admin 2 units in Uganda).

Travel time to nearest city in stage 3a revealed large variations in connectivity across Africa, contrasting the densely populated and urbanised regions in northern and western Africa with the Sahara and the rainforests of middle Africa. For instance, the isolation of forested areas in middle Africa contributed to substantial reductions in rankings (figure 2, third row, stage 3a), and suggest lower potential for a widespread outbreak of Crimean–Congo haemorrhagic fever, Ebola virus disease, or Marburg virus disease occurring in Congo (14% and 15% reduction in top quintile admin 2 for Ebola virus disease and Marburg virus disease), the Democratic Republic of the Congo (−43% Crimean–Congo haemorrhagic fever, −21% Ebola virus disease, −18% Marburg virus disease), and Gabon (−40% Ebola virus disease, −29% Marburg virus disease). Conversely, much of western Africa rose in the ranking for epidemic potential, particularly in Nigeria (+20% Crimean–Congo haemorrhagic fever, +29% Ebola virus disease, and +19% Marburg virus disease), Guinea (+28% Ebola virus disease, +27% Marburg virus disease), and Sierra Leone (+25% Ebola virus disease). A similar trend was seen for densely populated regions of eastern Africa, such as Uganda (+19% Ebola virus disease, +23% Marburg virus disease), indicating that these locations have a greater potential to spread to neighbouring areas. To that end, comparison of local outbreak potential with international spread capability also highlights important trends (figure 2, fourth row, stage 3b). For Crimean–Congo haemorrhagic fever, with only low rankings in receptivity, the potential for international spread was high for South Africa (81% increase in top quintile admin 2) and at-risk districts in Nigeria represented many of those with the highest potential for global spread (+30% Crimean–Congo haemorrhagic fever, +59% Ebola virus disease, +42% Marburg virus disease, +18% Lassa fever).

## Discussion

We report large heterogeneities across Africa in potential for zoonotic spillover resulting in index cases and their subsequent potential to result in local outbreaks or spread to neighbouring districts or countries. Several locations rank highly for localised outbreaks, but are comparatively less likely to spread elsewhere due to their isolation (eg, forests in middle Africa). Other locations, many in western Africa, rank highly both in terms of viral haemorrhagic fever outbreak potential and capacity to spread in the absence of effective interventions. Identification of regions with the greatest increases in rank between stages highlights key transition points in which interventions are crucial for preventing epidemics.

We hope this work can inform investments at each stage of potential epidemic progression: identification of regions requiring heightened surveillance in people and animals (stage 1) such as bat surveillance in Cameroon;^59^ strengthening of core pandemic preparedness and response capacities (stage 2) by addressing weaknesses in health provisioning in Chad, Somalia, and South Sudan; and pre-emptive identification of places that are likely to be key distributors in any potential outbreak (stage 3) such as highly connected Nigerian and Ugandan admin 2 units. For stage 1, this assessment highlights the subnational variation that exists in at-risk populations, providing additional evidence concerning spillover potential in places that are considered non-endemic, such as Ebola virus disease in the Central African Republic. Proactive surveillance in animal populations can help these locations better evaluate emerging infectious disease threats. Additionally, highest ranked locations should be provisioned with the necessary diagnostic capacity to ensure timely and accurate diagnosis.

Stage 2, quantifying outbreak potential, provides a data-informed framework for targeting where to focus resources for health-system strengthening. Coupling these with Joint External Evaluations to establish where there are unmet needs in outbreak response will identify key priorities for future investment. Ensuring health-care workers are aware of possible index-case presentation is an important first step in minimising the potential for nosocomial secondary spread.^60^ With candidate vaccines in development, these assessments can also inform where stockpiles might be most appropriate.^61^ Finally, stage 3, assessing the potential for further geographic spread, allows for an in-depth examination of where high connectivity could result in widespread infection and how to design interventions, such as ring vaccination,^62^ to halt continued transmission. Overlaying highly ranked locations identifies where these efforts could be most effective across a variety of scenarios (supplementary materials).

This framework is not dependent on there being an outbreak and can therefore support the proactive development of national and regional contingency plans by ministries of health and non-governmental organisations. Where vulnerabilities are identified, communities can appropriately prepare and rectify issues in advance. Understanding where potential outbreaks might occur and prospective transmission trajectories is an important step in establishing informed protocols for prevention and control. Indeed, even when cases occur, this framework combined with specific modelling strategies by stage and pathogen can inform focal control and response efforts. Aligning future priority pathogens with strategic investment in vaccine countermeasures targets (eg, Nipah virus) or other organisations' aims, will maximise the value of these outputs.

It is important to consider the limitations of this approach. The geographic scope of currently measured factors and the need for their completeness necessitates the use of proxy covariate factors rather than specific drivers. Assuming that spillover potential scales with population, rather than explicit assessment of human-animal interactions, was necessary since systematically collected data on these interactions and their relative frequencies were not available. Data gaps in emerging infectious disease epidemiology and transmission still remain, particularly regarding reservoirs. This flexible framework could, however, add new information such as quantitative measures of risk factors,^63^, ^64^ or include alternative modelling approaches^65^ where appropriate, when such assessments become more geographically comprehensive. Importantly, uncertainty at all stages can be propagated and included in estimates. To maximise generalisability, we were unable to include some pathogen-specific countermeasures. For instance, the high rankings of districts in Uganda and the Democratic Republic of the Congo do not account for historical viral haemorrhagic fever outbreaks in these locations^23^ and the sophisticated response systems now present, which will help offset some of the outbreak potential identified by this framework. Such response blueprints or experiences, however, can be referenced and replicated by other at-risk countries, including pre-emptive surveys of bat populations in Ghana,^66^ strategies to overcome logistical surveillance challenges used in Uganda,^60^ or recognition of where existing infrastructure could be co-opted, such as polio teams in Nigeria.^67^

Use of rankings versus absolute values meant that although comparative statements can be made, definitive statements of risk are harder. Given the evidence base currently available, we are able to identify potential spillover locations in a data-driven manner; however, such statements are not possible for stage 2 and 3 in the absence of more outbreak-specific information. For future iterations, parameterisation of outbreak receptivity using historical outbreaks could allow for this, and could also be used to weight the relative effect of factors. By leveraging the full history of outbreaks, these data will also provide a mechanism for formally validating the framework. When expanding to new regions, this ability to parameterise differences in factors will allow for regional variations to be considered. Rankings are also sensitive to estimated parameters such as the suitability thresholds defining risk at stage 1, which leads to inconsistent at-risk admin 2 level definitions based on varying thresholds (supplementary materials). Such variation is incorporated and reflected in broader uncertainty intervals. In spite of this fact, however, districts such as Guéckédou (the epicentre of the western African Ebola virus disease outbreak) remain in the 98th percentile of districts with high epidemic potential for Ebola virus disease, indicating that this approach can indeed provide valuable information on epidemic potential.

The appropriateness of factors in approximating for these processes is open to debate, as a trade-off between the geographic completeness and epidemiological relevance of data that was needed for inclusion. For instance, more appropriate proxies for local expenditure on health care beyond percentage of total gross domestic product could be leveraged as this information becomes increasingly available.^68^ Similarly, response time might not be solely dependent on travel infrastructure being present, but also its quality and year-round availability. With continued efforts to improve existing data resources or use geostatistical approaches to increase their spatial resolution,^69^, ^70^ these improved covariates can all be included in future iterations. Increasing the number of subnational covariates will also result in greater variation between districts within a country (supplementary materials), and is therefore particularly important when considering using such approaches to evaluate heterogeneity within a specific country. Additional pathogens will require other covariates to be considered. For instance, existing niche mapping approaches have been restricted to those pathogens strongly affected by environmental factors, whereas additional pathogens will necessitate quantification of an increasing number of socioeconomic factors.^71^, ^72^ The modular nature of this approach, however, allows for their inclusion should they become available.

Pathogens will continue to emerge and providing a rational and informed mechanism for the identification and prioritisation of regions for improvement and assistance across the world is therefore crucial. As demonstrated with African viral haemorrhagic fevers, this framework presents pandemic potential as distinct, yet interconnected stages, and allows for specific interventions to be tailored to locations where it is likely to have the greatest effect before the next epidemic. In moving to a more proactive consideration of these pathogens and regional variation in vulnerabilities, this analysis complements global health security ambitions and contributes to the larger discussion of where to focus limited resources in preparation for an epidemic of these rare, yet potentially devastating, pathogens.
